# Systematic Literature Review on Economic Evaluations and Health Economic Models in Metastatic Castration-Sensitive Prostate Cancer

**DOI:** 10.3390/curroncol32080412

**Published:** 2025-07-22

**Authors:** Thanh Tu Nguyen, David Ameyaw, George Dennis Obeng, Rose Amuah, Judit Józwiak-Hagymásy, Tamás Dóczi, Dóra Mezei, Bertalan Németh, Attila Tordai, Ahu Alanya, Guillaume Grisay, Marcell Csanádi

**Affiliations:** 1 Center for Health Technology Assessment, Semmelweis University, 1091 Budapest, Hungary; 2 Center for Pharmacology and Drug Research & Development, Semmelweis University, 1085 Budapest, Hungary; 3 Syreon Research Institute, 1145 Budapest, Hungary; 4 Syreon Research Africa, Accra F892/1, Ghana; 5 Department of Transfusion Medicine, Semmelweis University, 1089 Budapest, Hungary; 6 Quality of Life Department, European Organization for Research and Treatment of Cancer (EORTC), 1200 Brussels, Belgium; 7 Department of Medical Oncology, Centres Hospitaliers Universitaires HELORA, 7100 La Louvière, Belgium

**Keywords:** prostate cancer, economic evaluation, health economic modelling, systematic literature review, evidence synthesis

## Abstract

At diagnosis, metastatic prostate cancer (PC) is sensitive to androgen deprivation therapy (ADT), and patients are usually referred to as having castration-sensitive prostate cancer (mCSPC). The combination of ADT and androgen receptor pathway inhibitors (ARPI) is the current standard of care for mCSPC. This study aimed to review the literature on economic evaluations and health economic models related to mCSPC. A literature search was performed covering Medline, Embase, and Scopus with additional grey literature sources. Studies with data on health economic evaluations focusing on Europe or North America were relevant. 18 peer-reviewed articles and 10 grey literature documents were included. The majority (*n* = 23) had a deterministic Markov structure and applied either Markov cohort or partitioned survival models. Evaluations investigated various types of ADT-based combinations, comparing the addition of ARPI, chemotherapy agents, or radiation therapy to ADT alone. We concluded that economic evaluations in the field of PC are widely published, and there are a large number of publications even in the specific subgroup of mCSPC. Regardless of the investigated interventions, most studies applied similar methodologies and simulated patients from the mCSPC state until the development of mCRPC or death.

## Simple Summary

Prostate cancer is one of the most common cancers among men. With budget constraints and the ever-growing innovation in treatments, health economic evaluations play a crucial role in decision-making. This systematic review aims to synthesize available economic evaluations and health economic models in the field of metastatic castration-sensitive prostate cancer treatments, with a specific focus on Europe and North America. This article focuses on the methodology and the modeling approach of the identified health economic evaluations. In general, the most common model type is the deterministic Markov model structure, including Markov cohort models or partitioned survival models. Patient pathways were simulated from castration-sensitive to castration-resistant and dead in most cases. Our findings could assist researchers and decision makers in rapid decision making in appropriate contexts and/or the development of future evaluations of treatments.

## 1. Introduction

Prostate cancer (PC) is the world’s second most common cancer and the fifth leading cause of cancer-related deaths among men, with an estimated 1.5 million new cases and 397,000 attributable deaths [1]. In the United States, PC is the second leading cause of cancer-related deaths among men, and since 2014, the incidence rate has increased 3% per year [2]. 313,708 new cases and 35,770 deaths have been estimated in 2025 [2]. In Europe, PC was the most diagnosed cancer (over 500,000 new cases), contributing to more than 100,000 annual cancer-related deaths among men [3].

Treatment for PC is generally dependent on the invasiveness of the disease. Patients with localized cancer may be placed on active surveillance if they have low-risk disease or treated with curative intent by either radiotherapy with or without androgen deprivation therapy (ADT) or radical prostatectomy [4]. High-risk metastatic disease is treated with ADT in combination with an androgen receptor pathway inhibitor (ARPI) or with the combination of chemotherapy and ARPI (“triplet” therapy) [5].

Patients diagnosed with metastatic disease often respond to androgen deprivation; these patients are usually referred to as metastatic castration-sensitive prostate cancer or metastatic hormone-sensitive prostate cancer (mCSPC or mHSPC) patients. However, as a result of hormonal therapy, eventually, all PC will advance into castration resistance, which is called metastatic castration-resistant prostate cancer or metastatic hormone-resistant prostate cancer (mCRPC or mHRPC). Patients with mCRPC may continue with intensified ADT in addition to several possible drugs, including radiopharmaceuticals and/or targeted therapies (poly ADP ribose polymerase-PARP inhibitor) and/or chemotherapy and/or ARPI if they were not treated with one in the mCSPC setting [5].

Until 2015, ADT was the standard of care for mCSPC patients, where the combination of ADT and an ARPI was indicated for mCRPC [6]. However, results from groundbreaking trials such as CHAARTED or STAMPEDE, followed by many others, led to the acceptance of the combination treatment in the mCSPC setting due to the observed significant improvement in the overall survival of patients [6,7,8]. On the other hand, despite the significant improvements in patient care, the continuous treatment of mCSPC with both ADT and an ARPI is accompanied by a potential negative impact on patients’ quality of life and increased resource consumption resulting mainly from the adverse events management and the associated costs [9].

With the scarcity of resources and the ever-growing innovation in healthcare, especially in cancer management, it is necessary to select interventions that offer the best value for money (whatever that value may be), whether choosing between different treatments for a disease or selecting a basket of interventions for a population’s health priorities [10]. Economic evaluation is a generally accepted approach to achieving this objective. Economic evaluations compare both costs and health outcomes of two or more alternative interventions to determine their cost-effectiveness or budget impact, usually against a threshold [11]. Economic modeling is the predominant method of economic evaluation, mostly accepted by the various stakeholders, given its ability to determine the future costs and consequences of a decision from various data sources and considering its ability to account for future uncertainties [10]. A number of economic modeling techniques exist (mainly decision tree, Markov, and discrete event simulation) suitable for varying contexts and decision problems, but none is without limitations, which can come from the simplification of the patient pathways, the representativeness of patient characteristics, or the limited availability of data for the modeling purposes.

Comprehensive reviews of economic evaluations and economic modeling in various fields of oncology are warranted to understand the trends of health economics evidence generation, identify evidence gaps, and supplement the existing analyses, which are focused on specific therapeutic interventions [12]. This is also essential to guide rapid decision making in appropriate contexts and/or the development of future economic evaluations for mCSPC treatments.

There are available systematic reviews on the economic evaluation of metastatic PC treatments that were limited to mCRPC [13,14] or had a mixed population with other disease subgroups as well [15,16,17,18,19]. Notably, the reviews focusing specifically on mCSPC are more limited. For instance, Pelloux-Prayer et al. studied economic evaluations for mCSPC but only included studies published between a short time period, 2016 and 2021 [20]. Yanev et al. reviewed cost-effectiveness studies for both mCSPC and mCRPC but only in large medical databases without looking at the grey literature sources [21].

This current study, therefore, aimed to comprehensively review available economic evaluations and health economic models related to mCSPC treatments, with a specific focus on Europe and North America.

## 2. Materials and Methods

The systematic literature search was performed covering the following databases: Medline (via PubMed), Embase, and Scopus, in September 2023. A review protocol was developed before the start of the work, and it is a component of the DE-ESCALATE project (Grant No. 101104574). The protocol follows a predefined review plan approved by the DE-ESCALATE Consortium. The PICOS criteria were applied to define the research questions (see Appendix A, Table A1). The original search was re-run for updating the review in November 2024. Search strings for each database were constructed to identify all possible studies that contained data related to health economic evaluation on the PC population from 2008. No restrictions on the intervention (i.e., treatment, diagnosis, or screening) or PC stage were applied at the phase of the literature search. Details about search strings and the number of hits in each database are presented in Appendix A, Table A2, Table A3 and Table A4. To increase the sensitivity of the systematic review, snowball searches were also conducted by checking the references of the included articles to find more relevant studies.

Since evidence not published in peer-reviewed journals can contribute to the comprehensiveness of a systematic review, grey literature sources were included in the search strategy. To find relevant health economic evaluations and models related to PC, relevant health technology assessment/appraisal agencies and conference abstracts were searched, which include the International Society for Pharmacoeconomics and Outcomes Research (ISPOR), the National Institute for Health and Care Excellence (NICE), and the Canadian Agency for Drugs and Technologies in Health (CADTH), among others (see the full list in Appendix A, Table A5).

All title and abstract screening, full-text screening, and data extraction were conducted by at least two researchers independently and reported in compliance with the PRISMA 2020 Statement [22]. In case of disagreement between the two independent researchers on inclusion/exclusion, the principal investigator was invited to decide on each case. We used Covidence for screening and Excel for data collection. No generative artificial intelligence (GenAI) has been used in this review study.

At the abstract screening phase, the articles were screened through the following exclusion criteria in a hierarchical order: (1) No English abstract and irrelevant title; (2) No human subjects in the scope of the study; (3) Prostate cancer patients are not in the focus of the study; (4) Case studies or case series; (5) Not related to health economic evaluations or models; (6) Economic evaluations or models not related to Europe or North America.

At the full-text screening, we included all health economic evaluations of PC treatments. The papers were screened through the following exclusion criteria in a hierarchical order: (1) Not focusing on prostate cancer patients; (2) Not reporting original data; (3) Not written in English; (4) Not health economic evaluations or models; (5) No relevant data—economic evaluation is mentioned without details; (6) Not related to curative treatment of primary disease; (7) Screening or diagnosis-related economic evaluation; (8) Not Europe or North America; (9) Former systematic literature reviews with a relevant topic.

From the identified studies, only those went under data extraction which specifically focused on mCSPC treatments. Data extraction was done using Microsoft Excel sheets. Besides basic study data, the focus of the data extraction was on the evaluation/modeling methodology applied by the included studies (e.g., type of economic evaluation, measure, patient population, model type, health states, model arms, country, perspective, time horizon, etc.). A narrative synthesis of the extracted data was performed. A summary of the findings is presented in the results section below.

Quality assessment of peer-reviewed articles was performed, where the methodological quality of the original publication was evaluated using the ECOBIAS checklist [23]. The 22-item checklist was developed to assess risk of bias in model-based economic evaluations, with questions focusing on bias related to structure, data, and consistency. Studies with limited information (i.e., conference posters) and materials from HTA agencies that were mostly appraisal documents with limited data on the original economic evaluations did not undergo the quality assessment.

## 3. Results

### 3.1. Literature Findings

**Figure 1:**
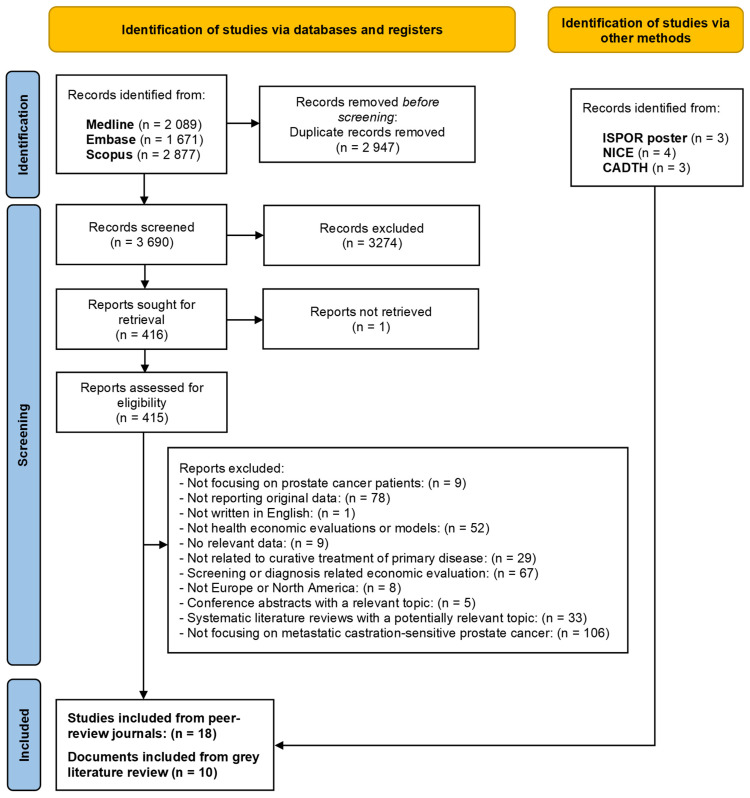
The PRISMA flow diagram of the literature search and selection process.

Our search resulted in the following hits in the different databases of publications: Medline—2089; EMBASE—1671; Scopus—2877. After importing these references to COVIDENCE, 3690 records were reviewed based on the title and abstract (automatically identified duplicates were 2947 records). The title and abstract screening excluded 3274 records and resulted in 416 potentially relevant records for full-text screening. Eventually, after full-text screening, 18 health economics evaluations on treatments of mCSPC patients were found and included in data extraction and quality assessment processes. Details are presented in the PRISMA flow diagram (Figure 1).

Regarding the grey literature, from the ISPOR database, we found 3 relevant conference posters, which did not overlap with the peer-reviewed publications. These conference posters were all published in 2023. From the NICE database, we found 4 relevant technology appraisal documents, while from the CADTH database, we found 3 relevant documents focusing on technology appraisal.

### 3.2. Health Economic Evaluations from Peer-Reviewed Publications

Among 18 studies with health economic evaluations on the treatment of mCSPC, there were 3 studies that used Markov simulation models [24,25,26], 1 study that used the hybrid model [27], 13 studies that used Markov cohort or partitioned survival models [28,29,30,31,32,33,34,35,36,37,38,39,40], and 1 study that did not use any traditional economic model types [41]. A summary of these health economic evaluations is presented in Table 1.

#### 3.2.1. Markov Simulation Models on mCSPC

Three included studies used Markov simulation models. Among them, two studies were conducted in North America (USA and Canada), and one study was performed in Europe (Italy). These models had lifetime horizons for the simulation of patient pathways, and they accounted for the risk of other causes of mortality.

Iannazzo [24] et al. performed a cost-effectiveness analysis to compare 5 formulations of LHRH agonists from the Italian National Health Service perspective. Importantly, the study did not consider the quality of life of patients, and the outcome measure was cost/life months gained. Clinical data was extracted from 129 metastatic PC patients in an Italian hospital and then analyzed in an economic model, using a patient-level microsimulation technique. The cost of LHRH agonists, follow-up examinations, and chemotherapy for the hormone-refractory phase were considered, except for the cost of toxicities of these treatments, as they assumed that it is the same across treatments.

Hird et al. investigated the cost-utility of ADT with initial docetaxel chemotherapy versus ADT with initial abiraterone acetate and prednisone in treating newly diagnosed mCSPC [25]. They developed a Markov microsimulation model from the perspective of a Canadian healthcare payer. The model included 5 health states: 1st-line treatment, which is with the investigated treatments; 2nd-line therapy; 3rd-line therapy; palliation; and death. Probabilities and utilities were calculated based on literature data. Short-term complications and inconvenience were taken into account as decrements in health state utility. The cost of treatments, emergency visits, hospitalizations, surveillance, adverse events, and palliation were considered.

Lester-Coll et al. analyzed the cost-utility of adding prostate radiation to ADT and ADT alone from the US payer perspective [26]. Microsimulation was used to follow and track individual events, such as treatment-related toxic effects. Especially grade 2 or higher genitourinary and gastrointestinal toxic effects were followed. The model had 4 health states: stable disease, progression, second progression, and death. Costs of radiotherapy toxicity were taken into account in the evaluation.

Quality assessment performed for the three studies that used the Markov simulation model found no major issues of concern. Sponsorship was transparently disclosed in 2 out of the 3 studies. The model structures were adequately chosen and in line with patient pathways, and the lifetime time horizon was appropriate. The methods of data identification were transparent. Utilities were appropriately incorporated for the decision problem in all studies except in Iannazzo, 2011 [24], where life years gained (LYG) was the primary measure of benefit. Uncertainty was analyzed in all studies. Two studies reported on some form of internal consistency checks, which is an important point for quality assurance [25,26]. Details of the quality assessment can be found in Appendix B, Table A6.

#### 3.2.2. Hybrid Economic Model mCSPC

There was only a study by Lu et al. that used a hybrid model, from the United Kingdom [27]. The study compared different types of ADTs (see Table 1). It was a combination of a decision tree and a Markov model. In the first month of the patient pathway, a decision tree model was used, and then for evaluating the long-term outcomes, a Markov model was constructed. The decision tree monitored patients from the start of hormonal treatment to the end of month 1. During this time, patients either developed complications (i.e., severe spinal cord compression, mild symptomatic spinal cord compression, or bladder outlet obstruction) or had no complications. It was assumed that all patients, regardless of complications, continue hormone treatment for PC. In the Markov model there were 3 health states: in response, in progressive disease, and dead. Besides the initial flare, side effects were assumed to be similar among treatments.

The study was of good quality upon assessment, with no major issue of concern. Details of the quality assessment can be found in Appendix B, Table A7.

#### 3.2.3. Markov Cohort Models/Partitioned Survival Models on mCSPC

There were 13 studies that used a deterministic Markov model structure, and these were conducted in the USA (7 studies), Canada (3 studies), Switzerland (1 study), Belgium (1 study), and France (1 study) [28,29,30,31,32,33,34,35,36,37,38,39,40]. These were either Markov cohort models or partitioned survival models. Importantly, it was not reported specifically for every model whether they had a Markov cohort type simulation or a partitioned survival approach. In these 6 cases, the specific approach was assumed based on the model design description in the article. In 9 cases a Markov cohort model design was used (assumed in 5 cases), while in 4 cases it was a partitioned survival model (assumed in 1 case). Details are presented in Table 1.

Most of the studies (10 out of 13) compared intensified ADT with ADT alone. Investigated intensified ADTs include the combination of ADT with the following medications: docetaxel, apalutamide, enzalutamide, abiraterone acetate plus prednisone, and darolutamide. The rest of the studies [30,31,33] compared intensified ADTs, ADT with other therapies, e.g., metastasis-directed therapy, surveillance, or with each other. There were especially 2 studies that compared the cost-effectiveness of different treatment sequencing strategies for intensified ADTs [31,33].

QALY was the most common outcome employed to measure the benefit of interventions. 11 out of 13 studies used QALYs. Additionally, one study also used progression-free quality-adjusted life years. Meanwhile, 3 studies used life years gained. Five studies had lifetime horizon models. Two studies applied a 15-year and 10-year ’time horizon. Besides, 30-year, 20-year, 5-year, and 3-year time horizons were also used.

The majority (9 out of 13) of these studies applied 3 “typical” health states, which are progression-free disease, progressive disease, and death. Besides, there were 4 studies that used different health states. Bleser et al. compared 3 treatment strategies: (1) metastasis-directed therapy with delayed ADT, (2) surveillance with delayed ADT, and (3) immediate ADT by applying a Markov trial-based model with 4 health states: (1) ADT-free, (2) ADT-state, (3) castration-resistant PC, and death [30]. In the study of Parikh et al., the authors used 4 health states: low-volume M1 disease stage, castrate-resistant disease, death from PC, and death from other causes [31]. In addition, the arm of metastasis-directed therapy had one more state, which is high-volume mHSPC. Moreover, the Markov model in the study of Ramamurthy et al. included stable health states with and without adverse events (i.e., fatigue, neutropenia, neutropenic fever) and disease progression, which also included death [34]. Additionally, a four-state Markov model was developed: mHSPC, mHRPC, prostate cancer death, and all-cause death in Sathianathen et al.′s analysis [36].

The clinical data to build the models in the studies was collected from many available relevant clinical trials, including the CHAARTED, the LATITUDE, the STAMPEDE, the ARCHES, the PEACE, the ARANSENS, the TITAN, or the ENZAMET trial.

Many types of costs were considered in the models, e.g., costs of treatments, costs of diagnostics, costs of follow-up, physician visits, nursing time, drug administration, laboratory testing, imaging, best supportive care, palliative radiotherapy, end-of-life care, one-off terminal hospitalization, clerical work, and cost of adverse events/toxicities. However, as none of the identified studies applied a societal perspective, no indirect costs were considered. Adverse events and toxicities are derived from clinical trials, literature, and expert opinion. In many models (10 out of 13), adverse events and toxicities were taken into account as utility decrements of health states.

Quality assessment was performed for these model-based economic evaluation studies. The overall quality of the assessed studies was good. A few important findings of the assessment are as follows. A single study did not apply discounting; however, the time horizon of the model was more than one year [30]. Sponsorship was transparently disclosed in most cases. The model structures were adequately chosen and in line with patient pathways. The time horizon was mostly 10+ years except in two studies [30,34], in which the authors justified a 3-year time horizon as the available follow-up for a landmark trial and a 5-year time horizon as the most appropriate extrapolation from a three-year trial due to limited data on survival, respectively. The methods of data identification were transparent. Utilities were appropriately incorporated for the decision problem except in Pelloux-Prayer, 2021, where life years gained (LYG) was the primary measure of benefit [33]. Uncertainty was variably analyzed in all studies. Only Wang (2022) reported on some form of internal consistency checks, which is an important point for quality assurance [39]. Details of the quality assessment can be found in Appendix B, Table A8.

### 3.3. Health Economic Evaluations from Conference Posters

We identified 3 health economic evaluations, which did not overlap with the formerly described publications and were presented at ISPOR congresses [42,43,44]. These evaluations assess the cost-utility of mHSPC treatments. All these studies used partitioned survival models over a lifetime horizon with 3 health states, which are commonly used in oncology: progression-free, progression, and death. They all measured cost per QALY as the primary outcome. Details are presented in Table 2.

### 3.4. Health Economic Evaluations from HTA Bodies

The review of the NICE database resulted in 4 relevant technology appraisal documents [45,46,47,48]. A summary of these documents is presented in Table 3. Notably, the modelling approach of these 4 documents included sub-health states for different lines of therapy in case of disease progression. In addition, two of these studies also had sub-health states for patients on treatment and off treatment. This latter approach was not observed in the former publications described above.

The review of the Canadian HTA agency website resulted in 3 additional technology appraisal documents [49,50,51]. However, 2 of them have limited information on the modelling approach. A brief summary of these documents is presented in Table 4.

## 4. Discussion

This systematic review reports a comprehensive overview of the current literature related to the economic evaluations of various treatment alternatives in mHSPC. We focused on the methodological aspects and the modelling approaches. A grey literature search and a snowball sampling were also conducted to enhance the comprehensiveness. Furthermore, quality assessment was carried out to identify potential biases that may be associated with the included studies. Eventually, the 18 peer-reviewed articles, the 3 conference posters, and the 7 documents from the HTA agencies provide an extensive picture about the current state of the art. As part of the review process, we also identified former literature reviews with similar scope. Among the 33 systematic reviews that were excluded at the full-text review phase, we identified two highly similar systematic reviews. These were both published in 2022 by Pelloux-Prayer et al. and Yanev et al. [20,21].

The review by Pelloux-Prayer is highly relevant to our work, as they also specifically focused on therapies for mHSPC patients [20]. This work was limited, though, in terms of the timeline, as they identified health economic evaluations between 2016 and 2021. With their global focus, they found 14 relevant evaluations; importantly, all 6 studies they found from Europe or North America were also found by our search. The review by Yanev focused on two subgroups of patients: mHSPC and non-metastatic castration-resistant prostate cancer [21]. They found 11 publications related to mHSPC populations. Importantly, all these publications with the focus on mHSPC patients in North America and Europe were also identified by our comprehensive search.

The majority of economic evaluations investigated intensified ADT treatment alternatives (combinations of ADT with ARPI, chemotherapy agents, or radiation therapy) with ADT alone. This was anticipated considering the fact that ADT alone was the standard of care for mCSPC patients until recent studies demonstrated significantly improved survival with ADT intensification. Interestingly, there were two studies that investigated the cost-effectiveness of different intensified ADT sequences [31,33]. On the other hand, there is no study that compared the cost-effectiveness of continuous ATD with intermittent ones, which demonstrated non-inferior overall survival with less cost and adverse events [52]. This approach is currently being investigated in the EORTC 2238 DE-ESCALATE pragmatic trial to compare intermittent ADT + ARPIs with continuous therapy [53]. This study is also unique because it includes a health economics analysis integrated from the early phase of the trial to assess the economic value of the intermittent therapy, supporting evidence-based policymaking. This approach could serve as a model for both small and large international studies, making country-specific economic model adaptation easier and enabling more comparable policy decisions beyond Europe and North America.

A recently published review paper focused on the results of the economic evaluations in the mHSPC patient population, which was not in the scope of this paper. They specifically investigated whether triplet therapy (ADT plus ARPI with chemotherapy) provides good value for money compared to ADT plus ARPI combinations [54]. Due to the limited head-to-head comparisons, it is not clearly demonstrated that the additional health gain related to triplet therapy is justifiable from the cost-effectiveness point of view. The authors of that review recommend that the current role of triplets should be reserved for specific subgroups of mHSPC, mainly in resource-rich settings [54].

In this current review, all but one of the studies included were model-based economic evaluations. The predominant modelling technique was the deterministic Markov model: the Markov cohort model or the partitioned survival model. Patient pathways in these models mostly complied with the natural development of the disease, where patients move from castration-sensitive to castration-resistant and then die. The most common health states used were progression-free (mCSPC), progression (mCRPC), and dead or similar. Sub-health states are also used within these health states in some models.

Few of the studies used life-year gained or life-month gained to measure patient outcomes, and cost/QALY was still the most common outcome used in the included studies. There was no study from a societal perspective; all the studies were from the healthcare system or payer perspective. Interestingly, in the two former systematic reviews by Pelloux-Prayer and Yanev, studies with a societal perspective were found (5 in the former one and 2 in the latter one). However, all of these analyses were done in China. The included studies in our review were from European countries, the USA, and Canada. The highest number of studies was from the USA. Importantly, there were no studies identified from the Central and Eastern European region.

The quality assessment exercise showed that the overall quality of the included economic evaluations was adequate, with a few concerns that could be addressed in future studies. The two most frequently identified issues were the lack of comprehensive sensitivity analysis and the lack of reporting on the evaluation of internal consistency.

Although this review was conducted to the highest standards as much as possible, there are certain areas that could be considered as a limitation. First, even though our study was guided by a prior review protocol, the study protocol was not published or registered. Secondly, we focused on only studies from Europe and North America. Notwithstanding, the scope is broad enough and will serve the needs of researchers and decision-makers who are interested in this geographical scope. Finally, our original database search ended in September 2023. Findings from the original search were presented at the ISPOR Europe 2024 Conference [55].

To provide more up-to-date results, we re-ran the original searches in November 2024 on PubMed using the previous search syntax. After screening the newly identified records, we found three new relevant articles [56,57,58] and no additional relevant systematic reviews. All three studies were performed from the perspective of a payer or health care system in the USA. These studies were highly similar to the ones we identified, and a brief summary is provided below

From a payer perspective in the USA, Handorf et al. investigated the cost-utility of two treatment sequencing strategies: a combination of ADT and docetaxel followed by ADT and abiraterone acetate versus a combination of ADT with abiraterone acetate followed by ADT and docetaxel [56]. Unlike the 2 studies that compared treatment sequencing we found previously [31,33], which used Markov cohort models, this study used a microsimulation approach to build a discrete-time state transition model to manage the complex possibilities of therapy switching. The model structure for the two lines of therapy includes 8 health states: Line 1, Adverse event line 1, Post-line 1, Line 2, Adverse event line 2, Post-line 2, Extensive disease, and Dead.

Sathianathen et al. conducted a cost-utility analysis to compare ADT intensification strategies with docetaxel and/or ARPI and ADT alone from the USA health sector perspective [57]. They developed a Markov state-transition model, which includes 4 health states: mHSPC, CRPC, PC-specific mortality, and all-cause mortality. In general, this study is highly similar to the previous ones we identified in terms of the treatments investigated and modeling method.

Kramer et al. studied the cost-utility of adding local prostate radiotherapy to the standard of care from the United States healthcare system perspective [58]. Unlike the study of Lester-Coll [26] (identified by our review), which used a Markov simulation model to investigate adding prostate radiation therapy to ADT, this study developed a partitioned survival model with three health states: failure-free, progressive disease, and dead. In general, the modeling approach is highly similar to the ones we identified in this review.

## 5. Conclusions

Our comprehensive literature review reported on the economic evaluations and their related economic modeling techniques on treatments for mCSPC in Europe and North America. We concluded that economic evaluations in the field of PC are widely published, and there are a large number of publications even in the specific subgroup of mCSPC. Our findings could be valuable to researchers and decision makers as a guide for rapid decision making in appropriate contexts and/or the development of future evaluations of treatments.

## Figures and Tables

**Table 1:** Summary of health economic evaluations of mHSPC treatments in peer-reviewed publications.

Ref.	Investigated Therapies	Modelling Approach	Study Outcome	Study Country	Time Horizon
Iannazzo, 2011 [24]	(1) Leuprorelin low dose vs. (2) Leuprorelin high dose vs. (3) Triptorelin vs. (4) Buserelin vs. (5) Goserelin	Markov simulation model Health states in a Markov chain: alive or dead. Identical patients were simulated through the five treatment branches	Cost/life-month gained	Italy	Lifetime
Hird, 2020 [25]	(1) ADT with initial docetaxel chemotherapy vs. (2) ADT with initial abiraterone acetate and prednisone	Markov simulation model Health states: 1st line, 2nd line, 3rd line, palliation, death Events: treatment-associated complications, treatment-related death, disease progression	Cost/QALY	Canada	Lifetime
Lester-Coll, 2021 [26]	(1) ADT + Prostate Radiation Therapy vs. (2) ADT alone	Markov simulation model Health states: stable disease after initial treatment; progression; 2nd progression; death	Cost/QALY	USA	37 months to mirror the trial + Lifetime
Lu, 2012 [27]	(1) Degarelix vs. (2) Triptorelin + short-term flutamide + cyproterone or bicalutamide	Hybrid model Decision tree: 1 month to capture treatment complications Markov health states: in response, in progressive disease, dead	Cost/QALY	United Kingdom	10 years
Barbier, 2022 [28]	(1) ADT + docetaxel vs. (2) ADT + abiraterone vs. (3) ADT + apalutamide vs. (4) ADT+ enzalutamide vs. (5) ADT alone	Markov cohort Health states: Progression-free disease, progressive disease, death	Cost/QALY	Switzerland	30 years
Bleser, 2020 [30]	(1) Metastasis-directed therapy with delayed ADT vs. (2) Surveillance with delayed ADT vs. (3) Immediate ADT	Markov cohort (assumed) Health states: ADT-free, ADT-state, castration-resistant prostate cancer, death	Cost/QALY	Belgium	5 years
Parikh, 2020 [31]	(1) Metastasis-directed therapy followed by AAP + ADT, followed by docetaxel + ADT vs. (2) AAP + ADT followed by ADT + docetaxel vs. (3) ADT + docetaxel followed by ADT + AAP	Markov cohort (assumed) Health states: Low-volume M1, High-volume mHSPC, castrate-resistant prostate cancer, death (prostate cancer), death (other)	Net Monetary Benefit	USA	10 years
Pelloux-Prayer, 2021 [33]	Asymptomatic or mildly symptomatic patients (1) ADT + AAP → ADT + enzalutamide vs. (2) ADT + AAP → ADT + docetaxel vs. (3) ADT + docetaxel → ADT + abiraterone vs. (4) ADT + docetaxel →ADT + enzalutamide Symptomatic patients: (1) ADT + AAP → ADT + docetaxel vs. (2) ADT + docetaxel → ADT + cabazitaxel vs. (3) ADT + docetaxel →ADT + docetaxel	Markov cohort (assumed) Health states: mHSPC, mHRPC, death	Cost/LYG	France	Lifetime
Ramamurthy, 2019 [34]	(1) ADT + Abiraterone acetate vs. (2) ADT + Docetaxel vs. (3) ADT alone	Markov cohort Model 1: stable disease without AE, stable disease with fatigue, stable disease treatment discontinuation, disease progression/death Model 2: stable disease with neutropenia, stable disease neutropenic fever, stable disease no AE, stable disease post-chemo disease, progression/death	Cost/progression-free quality-adjusted life years	USA	3 years
Saad, 2022 [35]	(1) ADT + Enzalutamide vs. (2) ADT + Apalutamide vs. (3) ADT alone	Markov cohort (assumed) Health states: mHSPC, mHRPC, death	Cost/QALY	Canada	15 years
Sathianathen, 2019 [36]	(1) ADT + Docetaxel vs. (2) ADT + Abiraterone vs. (3) ADT alone	Markov cohort Health states: mHSPC, mHRPC, prostate-cancer death, all-cause death	Cost/QALY	USA	Lifetime
Sung, 2021 [37]	(1) ADT + Docetaxel vs. (2) ADT + Abiraterone vs. (3) ADT + Enzalutamide vs. (4) ADT + Apalutamide vs. (5) ADT alone	Markov cohort Health states: progression-free, progression, death	Cost/QALY	USA & China	Lifetime
Zhang, 2021 [40]	(1) Enzalutamide + ADT vs. (2) ADT alone	Markov cohort (assumed) Health states: Progression-free survival, progressive disease, death	Cost/QALY	USA & China	20 years
Beca, 2019 [29]	(1) ADT + docetaxel vs. (2) ADT alone	Partitioned survival model Health states: mHSPC, mHRPC, death	Cost/QALYCost/LYG	Canada	15 years
Parmar, 2022 [32]	(1) ADT + Apalutamide vs. (2) ADT alone	Partitioned survival model (assumed) Health states: Progression-free, progressive disease, death	Cost/QALYCost/LYG	Canada	Lifetime
Yoo, 2023 [38]	(1) ADT + Docetaxel vs. (2) ADT + AAP vs. (3) ADT + Apalutamide vs. (4) ADT + Enzalutamide vs. (5) ADT + Darolutamide and Docetaxel vs. (6) ADT + Enzalutamide and Docetaxel vs. (7) ADT alone	Partitioned survival model Health states: Progression-free, progression to mHRPC, death	Cost/QALY	USA	10 years
Wang, 2022 [39]	(1) ADT + docetaxel vs. (2) ADT + AAP vs. (3) ADT + enzalutamide vs. (4) ADT + apalutamide vs. (5) ADT alone	Partitioned survival model Health states: mHSPC, mHRPC, death	Cost/QALY	USA	Lifetime
Esteban, 2017 [41]	(1) ADT + docetaxel vs. (2) ADT alone	Incremental drug costs were divided by overall survival increment based on the CHAARTED and STAMPEDE studies	Cost/LYG	Spain	Non-applicable

ADT: androgen deprivation therapy; AAP: abiraterone acetate plus prednisone QALY: quality-adjusted life years; LYG: life year gain; mHSPC: metastatic hormone-sensitive prostate cancer; mHRPC: metastatic hormone-resistant prostate cancer; AE: adverse events.

**Table 2:** Summary of economic evaluations identified in conference materials.

**Ref.**	**Investigated Therapies**	**Modelling Approach**	**Study Outcome**	**Study Country**	**Time Horizon**
Katta, 2023 [42]	(1) ADT + Enzalutamide vs. (2) ADT + Apalutamide vs. (3) ADT + Abiraterone acetate	Partitioned survival model Progression-free, Progressed disease, Death	Cost/QALY	USA	Lifetime
Madani, 2023 [43]	(1) ADT Abiraterone acetate + Prednisone vs. (2) ADT alone	Partitioned survival model Pre-progression, Post-progression, Dead	Cost/QALY	UK	Lifetime
Nwogu, 2023 [44]	(1) ADT + Darolutamide + Docetaxel vs. (2) ADT + Docetaxel	Partitioned survival model Progression-free, Progressed disease, Death	Cost/QALY	USA	Lifetime

ADT: androgen deprivation therapy; QALY: quality-adjusted life years.

**Table 3:** Technology appraisal documents identified in the NICE database.

References	Investigated Therapies	Modelling Approach	Study Outcome	Time Horizon
TA903, 2023 [45]	(1) ADT + Darolutamide + docetaxel vs. (2) ADT + Docetaxel vs. (3) ADT + Enzalutamide vs. (4) ADT alone	Partitioned survival model Pre-progression with 2 sub-health states: on treatment, off treatment; Post-progression (3 sub-health states: 1st, 2nd, 3rd line); death	Cost/QALY	Lifetime
TA741, 2021 [46]	(1) ADT + Apalutamide vs. (2) ADT + Docetaxel vs. (3) ADT alone	Partitioned survival model Progression-free, progressed disease (3 sub-health states: 1st, 2nd, 3rd line), death	Cost/QALY	Lifetime
TA721, 2021 [47]	(1) ADT + Abiraterone acetate plus prednisone vs. (2) ADT + Docetaxel vs. (3) ADT alone	Partitioned survival model mHSPC (progression-free), mHSPC (progressive disease), mCRPC 1st, 2nd, 3rd line, death	Cost/QALY	20 years
TA712, 2021 [48]	(1) ADT + Enzalutamide vs. (2) ADT + Docetaxel vs. (3) ADT alone	Partitioned survival model Stable disease (with 2 sub-health states: on treatment, off treatment), progressed disease (3 sub-health states: 1st, 2nd, 3rd line), death	Cost/QALY	Lifetime

ADT: androgen deprivation therapy; QALY: quality-adjusted life years; LHRH: luteinizing hormone-releasing hormone; mHSPC: metastatic hormone-sensitive prostate cancer; mCRPC: metastatic castration-resistant prostate cancer.

**Table 4:** Technology appraisal documents identified in the CADTH database.

References	Investigated Therapies	Modelling Approach	Study Outcome	Time Horizon
PC0294-000 [49]	(1) ADT + Darolutamide + docetaxel vs. (2) ADT vs. Docetaxel vs. (3) ADT + Abiraterone and prednisone vs. (4) ADT + Apalutamide vs. (5) ADT + Enzalutamide vs. (6) ADT alone	Partitioned survival model mCSPC, mCRPC, death	Cost/QALY	Lifetime
PC0209-000 [50]	(1) ADT + Enzalutamide vs. (2) ADT + Docetaxel vs. (3) ADT + Apalutamide vs. (4) ADT + Abiraterone acetate plus prednisone vs. (5) ADT alone	Markov cohort No mention about health states	Cost/QALY	15 years
PC0200-000 [51]	(1) ADT + Apalutamide vs. Docetaxel + ADT vs. (2) ADT + Abiraterone acetate plus prednisone vs. (3) ADT alone	Partitioned survival model No mention about health state	Cost/QALY	20 years

ADT: androgen deprivation therapy; QALY: quality-adjusted life years; mHSPC: metastatic hormone-sensitive prostate cancer; mCRPC: metastatic castration-resistant prostate cancer.

## Data Availability

Systematic data collection was performed for the review. Data that were collected during the review but not presented in this article are available upon request. Further inquiries can be directed to the corresponding author.

## Abbreviations

The following abbreviations are used in this manuscript:

ADT	Androgen Deprivation Therapy
ARPI	Androgen Receptor Pathway Inhibitors
CADTH	Canadian Agency for Drugs & Technologies in Health
GenAI	Generative Artificial Intelligence
HTA	Health Technology Assessment
ISPOR	International Society for Pharmacoeconomics and Outcomes Research
LHRH	Luteinizing hormone-releasing hormone
LYG	Life Years Gained
mCRPC	Metastatic Castration-Resistant Prostate Cancer
mCSPC	Metastatic Castration-Sensitive Prostate Cancer
mHRPC	Metastatic Hormone-Resistant Prostate Cancer
mHSPC	Metastatic Hormone-Sensitive Prostate Cancer
NICE	National Institute for Health and Care Excellence
PC	Prostate Cancer
PRISMA	Preferred Reporting Items for Systematic Reviews and Meta-Analyses
QALY	Quality-Adjusted Life-Year

## Appendix A

**Table A1:** PICOS criteria to define the research questions.

P (patient/population)	patients with prostate cancer
I (intervention/indicator)	all systemic treatment patterns (studies on screening or diagnosis will be excluded)
C (comparison)	all systemic treatment patterns (studies on screening or diagnosis will be excluded)
O (outcomes of interest)	data related to health economic evaluations and models
S (study design/setting)	cost-effectiveness analyses cost-benefit analyses cost-utility analyses health economic analyses health economic evaluations health technology assessments

**Table A2:** Search string and number of hits in Medline.

Search No.	Concepts	Search String
#1	Patient	(“prostate”[Title/Abstract] OR “prostatic”[Title/Abstract]) AND (“cancer”[Title/Abstract] OR “neoplasm”[Title/Abstract] OR “tumor”[Title/Abstract] OR “tumour”[Title/Abstract])
#2	Outcome	(cost[Title/Abstract] AND effectiveness[Title/Abstract]) OR cost-effectiveness[Title/Abstract] OR “cost-effectiveness analys*”[Title/Abstract] OR cea[Title/Abstract] OR (cost[Title/Abstract] AND benefit[Title/Abstract]) OR cost-benefit[Title/Abstract] OR “cost-benefit analys*”[Title/Abstract] OR cba[Title/Abstract] OR (cost[Title/Abstract] AND utility[Title/Abstract]) OR cost-utility[Title/Abstract] OR “cost-utility analys*”[Title/Abstract] OR cua[Title/Abstract] OR “incremental cost effectiveness ratio”[Title/Abstract] OR “incremental cost-effectiveness ratio”[Title/Abstract] OR icer[Title/Abstract] OR “discrete event simulation”[Title/Abstract] OR markov[Title/Abstract] OR “decision tree”[Title/Abstract] OR (economic[Title/Abstract] AND (model*[Title/Abstract] OR analys*[Title/Abstract] OR evaluation[Title/Abstract])) OR “health technology assessment”[Title/Abstract] OR hta[Title/Abstract] OR ((health[Title/Abstract] AND economic[Title/Abstract]) AND (model*[Title/Abstract] OR analys*[Title/Abstract] OR evaluation[Title/Abstract])) OR “event history model”[Title/Abstract] OR microsimulation[Title/Abstract] OR “Monte Carlo”[Title/Abstract] OR “Monte-Carlo”[Title/Abstract] OR “patient level simulation”[Title/Abstract] OR “patient-level simulation”[Title/Abstract] OR “deterministic sensitivity analys*”[Title/Abstract] OR “probabilistic sensitivity analys*”[Title/Abstract] OR “simulation model*”[Title/Abstract] OR “transition probabilit*”[Title/Abstract] OR “Tornado diagram”[Title/Abstract]
#3	Combined search	#1 AND #2
#4	Filters	Limit to 2008–2023
#5	Filters	Limit to English (2089 hits)

**Table A3:** Search string and number of hits in Embase.

Search No.	Concepts	Search String
#1	Patient	(prostate:ti,ab,kw OR prostatic:ti,ab,kw) AND (cancer:ti,ab,kw OR neoplasm:ti,ab,kw OR tumor:ti,ab,kw OR tumour:ti,ab,kw)
#2	Outcome	cost:ti,ab,kw AND effectiveness:ti,ab,kw OR ‘cost effectiveness’:ti,ab,kw OR ‘cost-effectiveness analys*’:ti,ab,kw OR cea:ti,ab,kw OR (cost:ti,ab,kw AND benefit:ti,ab,kw) OR ‘cost benefit’:ti,ab,kw OR ‘cost-benefit analys*’:ti,ab,kw OR cba:ti,ab,kw OR (cost:ti,ab,kw AND utility:ti,ab,kw) OR ‘cost utility’:ti,ab,kw OR ‘cost-utility analys*’:ti,ab,kw OR cua:ti,ab,kw OR ‘incremental cost effectiveness ratio’:ti,ab,kw OR ‘incremental cost-effectiveness ratio’:ti,ab,kw OR icer:ti,ab,kw OR ‘discrete event simulation’:ti,ab,kw OR markov:ti,ab,kw OR ‘decision tree’:ti,ab,kw OR (economic:ti,ab,kw AND (model*:ti,ab,kw OR analys*:ti,ab,kw OR evaluation:ti,ab,kw)) OR ‘health technology assessment’:ti,ab,kw OR hta:ti,ab,kw OR (health:ti,ab,kw AND economic:ti,ab,kw AND (model*:ti,ab,kw OR analys*:ti,ab,kw OR evaluation:ti,ab,kw)) OR ‘event history model’:ti,ab,kw OR microsimulation:ti,ab,kw OR ‘monte carlo’:ti,ab,kw OR ‘monte-carlo’:ti,ab,kw OR ‘patient level simulation’:ti,ab,kw OR ‘patient-level simulation’:ti,ab,kw OR ‘deterministic sensitivity analys*’:ti,ab,kw OR ‘probabilistic sensitivity analys*’:ti,ab,kw OR ‘simulation model*’:ti,ab,kw OR ‘transition probabilit*’:ti,ab,kw OR ‘tornado diagram’:ti,ab,kw
#3	Combined search	#1 AND #2
#4	Filters	Limit to 2008–2023
#5	Filters	Limit to English
#6	Filters	Limit to article (1067 hits)

**Table A4:** Search string and number of hits in Scopus.

Search No.	Concepts	Search String
#1	Patient	TITLE-ABS-KEY (prostate OR prostatic) AND (cancer OR neoplasm OR tumor OR tumour)
#2	Outcome	TITLE-ABS-KEY ((cost AND effectiveness) OR cost-effectiveness OR “cost-effectiveness analys*” OR cea OR (cost AND benefit) OR cost-benefit OR “cost-benefit analys*” OR cba OR (cost AND utility) OR cost-utility OR “cost-utility analys*” OR cua OR “incremental cost effectiveness ratio” OR “incremental cost-effectiveness ratio” OR icer OR “discrete event simulation” OR markov OR “decision tree” OR (economic AND (model* OR analys* OR evaluation)) OR “health technology assessment” OR hta OR ((health AND economic) AND (model* OR analys* OR evaluation)) OR “event history model” OR microsimulation OR “Monte Carlo” OR “Monte-Carlo” OR “patient level simulation” OR “patient-level simulation” OR “deterministic sensitivity analys*” OR “probabilistic sensitivity analys*” OR “simulation model*” OR “transition probabilit*” OR “Tornado diagram”)
#3	Combined search	#1 AND #2
#4	Filters	Limit to 2008–2024
#5	Filters	Limit to English
#6	Filters	Limit to subject area: medicine
#7	Filters	Limit to article (2877 hits)

**Table A5:** List of grey literature sources.

Grey Literature Sources
Agency for Healthcare Research and Quality (AHRQ) Canadian Agency for Drug and Technologies in Health (CADTH) National Institute for Health and Care Excellence (NICE) Medical Services Advisory Committee (MSAC) Ludwig Boltzmann Institut für Health Technology Assessment (LBI) Belgian Health Care Knowledge Centre (KCE) Danish Health and Medicines Authority (DHMA) French National Authority for Health (HAS) Institut für Qualität und Wirtschaftlichkeit im Gesundheitswesen (IQWiG) National Centre for Pharmacoeconomics, Ireland (NCPE) National Health Care Institute Netherlands Norwegian Institute of Public Health (NIPH) Swedish Agency for Health Technology Assessment (SBU)

## Appendix B

**Table A6:** Summary Quality Assessment Results for Markov Simulation Model Studies.

Type of bias	Issues addressed	Iannazzo, 2011 [24]		Hird, 2020 [25]		Lester-Coll, 2021 [26]	
		Relevant to study Yes/No/Partly/Unclear/NA	How did you deal with this bias? (description of strategy and rationale)	Relevant to study Yes/No/Partly/Unclear/NA	How did you deal with this bias? (description of strategy and rationale)	Relevant to study Yes/No/Partly/Unclear/NA	How did you deal with this bias? (description of strategy and rationale)
PART A. Overall checklist for bias in economic evaluation							
Narrow perspective bias	Was a societal perspective adopted? If not, has a different perspective been justified?	Yes	Italian National Health Service perspective	Yes	healthcare payer perspective	Yes	US payer perspective
Inefficient comparator bias	Was the best alternative chosen as comparator? Was current practice chosen as a comparator? Have all comparators been described in sufficient detail?	Yes		Yes		Yes	
Cost measurement omission bias	Were all costs relevant to the disease and intervention identified and considered?	Yes		Yes		Yes	
Intermittent data collection bias	Was the resource use measured continuously?	Yes		Yes		partly	
Invalid valuation bias	Is the price calculation presented in a detailed manner? Have reference prices been used?	Yes		Yes		Yes	
Ordinal ICER bias	Have cardinal scales for the outcomes measure in a CEA been used?	Yes		Yes	cost/QALY	Yes	cost/QALY
Double-counting bias	Are variables adequately checked for double-counting?	Yes		Yes			
Inappropriate discounting bias	Have discounting rates from guidelines been applied?	Yes	3.50%	Yes	1.50%	Yes	3%
Limited sensitivity analysis bias	Have the four principles of uncertainty (methodological, structural, heterogeneity, and parameter) been considered in sufficient detail?	Partly	PSA	partly	One-way sensitivity analyses	Yes	PSA, DSA, scenarios
Sponsor bias	Have sponsorships been disclosed? Is the study protocol freely accessible?	Partly		partly		Unclear	
Reporting and dissemination bias	Has the study/trial been listed in a trial register? Have all results been reported according to the study protocol?	Unclear		Unclear		Unclear	
PART B. Model-specific aspects of bias in economic evaluation							
I Bias related to structure							
Structural assumptions bias	Is the model structure in line with coherent theory? Do treatment pathways reflect the nature of disease?	Yes		Yes		Yes	
No treatment comparator bias	Is there an adequate comparator, i.e., care as usual?	Yes		Yes		Yes	
Wrong model bias	Is the model chosen adequate regarding the decision problem?	Yes		Yes		Yes	
Limited time horizon bias	Was a lifetime horizon chosen? Were shorter time horizons adequately justified?	Yes		Yes	Lifetime	Yes	Lifetime
II Bias related to data							
Bias related to data identification	Are the methods of data identification transparent? Are all choices justified adequately? Do the input parameters come from high-quality and well-designed studies?	Yes		yes		Yes	
Bias related to baseline data	Are probabilities, for example, based on natural history data? Is transformation of rates into transition probabilities done accurately?	Unclear		yes		Yes	
Bias related to treatment effects	Are relative treatment effects synthesized using appropriate meta-analytic techniques? Are extrapolations documented and well justified? Are alternative assumptions explored regarding extrapolation?	Yes		partly		Yes	
Bias related to quality-of-life weights (utilities)	Are the utilities incorporated appropriate for the specific decision problem?	N/A	LYG used, not QALY	Yes		Yes	
Non-transparent data incorporation bias	Is the process of data incorporation transparent? Are all data and their sources described in detail?	Yes		yes		Yes	
Limited scope bias	Have the four principles of uncertainty (methodological, structural, heterogeneity, and parameter) been considered?	Partly		partly		Yes	
III Bias related to consistency							
Bias related to internal consistency	Has internal consistency in terms of mathematical logic been evaluated?	Unclear		yes		Yes	

**Table A7:** Summary Quality Assessment Results for Hybrid Model Study.

Type of bias	Issues addressed	Lu, 2012 [27]	
		Relevant to study Yes/No/Partly/Unclear/NA	How did you deal with this bias? (description of strategy and rationale)
PART A. Overall checklist for bias in economic evaluation			
Narrow perspective bias	Was a societal perspective adopted? If not, has a different perspective been justified?	Yes	UK NHS perspective
Inefficient comparator bias	Was the best alternative chosen as a comparator? Was current practice chosen as a comparator? Have all comparators been described in sufficient detail?	Yes	
Cost measurement omission bias	Were all costs relevant to the disease and intervention identified and considered?	Yes	
Intermittent data collection bias	Was the resource use measured continuously?	Yes	
Invalid valuation bias	Is the price calculation presented in a detailed manner? Have reference prices been used?	Yes	
Ordinal ICER bias	Have cardinal scales for the outcome measure in a CEA been used?	Yes	cost/QALY
Double-counting bias	Are variables adequately checked for double-counting?	Yes	
Inappropriate discounting bias	Have discounting rates from guidelines been applied?	Yes	3.50%
Limited sensitivity analysis bias	Have the four principles of uncertainty (methodological, structural, heterogeneity, and parameter) been considered in sufficient detail?	Yes	PSA and DSA done
Sponsor bias	Have sponsorships been disclosed? Is the study protocol freely accessible?	partly	
Reporting and dissemination bias	Has the study/trial been listed in a trial register? Have all results been reported according to the study protocol?	Unclear	
PART B. Model-specific aspects of bias in economic evaluation			
I Bias related to structure			
Structural assumptions bias	Is the model structure in line with coherent theory? Do treatment pathways reflect the nature of disease?	Yes	
No treatment comparator bias	Is there an adequate comparator, i.e., care as usual?	Yes	
Wrong model bias	Is the model chosen adequate regarding the decision problem?	Yes	
Limited time horizon bias	Was a lifetime horizon chosen? Were shorter time horizons adequately justified?	Yes	10 years
II Bias related to data			
Bias related to data identification	Are the methods of data identification transparent? Are all choices justified adequately? Do the input parameters come from high-quality and well-designed studies?	Partly	
Bias related to baseline data	Are probabilities, for example, based on natural history data? Is transformation of rates into transition probabilities done accurately?	Yes	
Bias related to treatment effects	Are relative treatment effects synthesized using appropriate meta-analytic techniques? Are extrapolations documented and well justified? Are alternative assumptions explored regarding extrapolation?	Partly	
Bias related to quality-of-life weights (utilities)	Are the utilities incorporated appropriate for the specific decision problem?	Yes	
Non-transparent data incorporation bias	Is the process of data incorporation transparent? Are all data and their sources described in detail?	Yes	
Limited scope bias	Have the four principles of uncertainty (methodological, structural, heterogeneity, and parameter) been considered?	Yes	
III Bias related to consistency			
Bias related to internal consistency	Has internal consistency in terms of mathematical logic been evaluated?	Unclear	

**Table A8:** Summary Quality Assessment Results for Deterministic Markov Cohort Model Studies.

Type of bias	Issues addressed	Barbier, 2022 [28]		Pelloux-Prayer, 2021 [33]		Ramamurthy, 2019 [34]		Saad, 2022 [35]		Sathianathen, 2019 [36]	
		Relevant to study Yes/No/Partly/Unclear/NA	How did you deal with this bias? (description of strategy and rationale)	Relevant to study Yes/No/Partly/Unclear/NA	How did you deal with this bias? (description of strategy and rationale)	Relevant to study Yes/No/Partly/Unclear/NA	How did you deal with this bias? (description of strategy and rationale)	Relevant to study Yes/No/Partly/Unclear/NA	How did you deal with this bias? (description of strategy and rationale)	Relevant to study Yes/No/Partly/Unclear/NA	How did you deal with this bias? (description of strategy and rationale)
PART A. Overall checklist for bias in economic evaluation											
Narrow perspective bias	Was a societal perspective adopted? If not, has a different perspective been justified?	Yes	Payer perspective	Yes	French public healthcare system perspective	Yes	US payer perspective	Yes	Canadian public payer perspective	Yes	US payer perspective
Inefficient comparator bias	Was the best alternative chosen as a comparator? Was current practice chosen as a comparator? Have all comparators been described in sufficient detail?	Yes		Yes		Yes		Yes		Yes	
Cost measurement omission bias	Were all costs relevant to the disease and intervention identified and considered?	Yes		Yes		Yes		Yes		Yes	
Intermittent data collection bias	Was the resource use measured continuously?	Yes		Yes		Unclear		Yes		Unclear	
Invalid valuation bias	Is the price calculation presented in a detailed manner? Have reference prices been used?	Yes		Yes		Partly		Yes		Yes	
Ordinal ICER bias	Have cardinal scales for the outcome measure in a CEA been used?	Yes	cost/QALY	Yes		Yes		Yes		Yes	
Double-counting bias	Are variables adequately checked for double-counting?	Yes		Yes		Yes		Yes		Unclear	
Inappropriate discounting bias	Have discounting rates from guidelines been applied?	Yes	3%	Yes	2.50%	No		Yes	1.50%	Yes	3%
Limited sensitivity analysis bias	Have the four principles of uncertainty (methodological, structural, heterogeneity, and parameter) been considered in sufficient detail?	Yes	PSA, DSA, many scenarios	Partly	DSA	Yes	PSA, DSA, and scenario analysis	Partly	PSA and scenario analysis	Yes	PSA, DSA, and scenario analysis
Sponsor bias	Have sponsorships been disclosed? Is the study protocol freely accessible?	partly		No		No		Partly		Partly	
Reporting and dissemination bias	Has the study/trial been listed in a trial register? Have all results been reported according to the study protocol?	Unclear		Unclear		Unclear		Unclear		Unclear	
PART B. Model-specific aspects of bias in economic evaluation											
I Bias related to structure											
Structural assumptions bias	Is the model structure in line with coherent theory? Do treatment pathways reflect the nature of disease?	Yes		Yes		Yes		Yes		Yes	
No treatment comparator bias	Is there an adequate comparator, i.e., care as usual?	Yes		Yes		Yes		Yes		Yes	
Wrong model bias	Is the model chosen adequate regarding the decision problem?	Yes		Yes		Yes		Yes		Yes	
Limited time horizon bias	Was a lifetime horizon chosen? Were shorter time horizons adequately justified?	Yes	30 years	Yes		Partly	3 years	Yes	15 years	Yes	Lifetime
II Bias related to data											
Bias related to data identification	Are the methods of data identification transparent? Are all choices justified adequately? Do the input parameters come from high-quality and well-designed studies?	Yes		Yes		Yes		Yes		Yes	
Bias related to baseline data	Are probabilities, for example, based on natural history data? Is transformation of rates into transition probabilities done accurately?	Yes		Yes		Yes		Yes		Yes	
Bias related to treatment effects	Are relative treatment effects synthesized using appropriate meta-analytic techniques? Are extrapolations documented and well justified? Are alternative assumptions explored regarding extrapolation?	Yes		Yes		Yes		Yes		Yes	
Bias related to quality of life weights (utilities)	Are the utilities incorporated appropriate for the specific decision problem?	Yes		N/A	Life years used, not QALY	Yes		Yes		Yes	
Non-transparent data incorporation bias	Is the process of data incorporation transparent? Are all data and their sources described in detail?	Yes		Yes		Yes		Yes		Yes	
Limited scope bias	Have the four principles of uncertainty (methodological, structural, heterogeneity, and parameter) been considered?	Yes		Partly		Yes		Partly		Yes	
III Bias related to consistency											
Bias related to internal consistency	Has internal consistency in terms of mathematical logic been evaluated?	Unclear		Unclear		Unclear		Unclear		Unclear	
Type of bias	Issues addressed	Sung, 2021 [37]		Wang, 2022 [39]		Yoo, 2023 [38]		Zhang, 2021 [40]			
		Relevant to study Yes/No/Partly/Unclear/NA	How did you deal with this bias? (description of strategy and rationale)	Relevant to study Yes/No/Partly/Unclear/NA	How did you deal with this bias? (description of strategy and rationale)	Relevant to study Yes/No/Partly/Unclear/NA	How did you deal with this bias? (description of strategy and rationale)	Relevant to study Yes/No/Partly/Unclear/NA	How did you deal with this bias? (description of strategy and rationale)
PART A. Overall checklist for bias in economic evaluation											
Narrow perspective bias	Was a societal perspective adopted? If not, has a different perspective been justified?	Yes	US payer perspective	Yes	US payer perspective	Yes	US public (Veterans Affairs) payer perspective	Yes	from the US and Chinese payers’ perspectives		
Inefficient comparator bias	Was the best alternative chosen as a comparator? Was current practice chosen as a comparator? Have all comparators been described in sufficient detail?	Yes		Yes		Yes		Yes			
Cost measurement omission bias	Were all costs relevant to the disease and intervention identified and considered?	Yes		Yes		Yes		Yes			
Intermittent data collection bias	Was the resource use measured continuously?	Unclear		Unclear		Unclear		Unclear			
Invalid valuation bias	Is the price calculation presented in a detailed manner? Have reference prices been used?	Yes		Yes		Yes		Yes			
Ordinal ICER bias	Have cardinal scales for the outcome measure in a CEA been used?	Yes		Yes		Yes		Yes			
Double-counting bias	Are variables adequately checked for double-counting?	Yes		Yes		Yes		unclear			
Inappropriate discounting bias	Have discounting rates from guidelines been applied?	Yes	3%	Yes	3%	Yes	3%	Yes	3% for China and 3.5% for US		
Limited sensitivity analysis bias	Have the four principles of uncertainty (methodological, structural, heterogeneity, and parameter) been considered in sufficient detail?	Yes	PSA, DSA, and scenario analysis	Yes	PSA and DSA	Partly	PSA and DSA	Partly	PSA and DSA done		
Sponsor bias	Have sponsorships been disclosed? Is the study protocol freely accessible?	No		Partly		Partly		Partly			
Reporting and dissemination bias	Has the study/trial been listed in a trial register? Have all results been reported according to the study protocol?	Unclear		Unclear		Unclear		Unclear			
PART B. Model-specific aspects of bias in economic evaluation											
I Bias related to structure											
Structural assumptions bias	Is the model structure in line with coherent theory? Do treatment pathways reflect the nature of disease?	Yes		Yes		Yes		Yes			
No treatment comparator bias	Is there an adequate comparator, i.e., care as usual?	Yes		Yes		Yes		Yes			
Wrong model bias	Is the model chosen adequate regarding the decision problem?	Yes		Yes		Yes		Yes			
Limited time horizon bias	Was a lifetime horizon chosen? Were shorter time horizons adequately justified?	Yes	Lifetime	Yes	Lifetime	Yes	10 years	Yes	20 years		
II Bias related to data											
Bias related to data identification	Are the methods of data identification transparent? Are all choices justified adequately? Do the input parameters come from high-quality and well-designed studies?	Yes		Yes		Yes		Yes			
Bias related to baseline data	Are probabilities, for example, based on natural history data? Is transformation of rates into transition probabilities done accurately?	Yes		Yes		Yes		Yes			
Bias related to treatment effects	Are relative treatment effects synthesized using appropriate meta-analytic techniques? Are extrapolations documented and well justified? Are alternative assumptions explored regarding extrapolation?	Yes		Yes		Yes		Yes			
Bias related to quality-of-life weights (utilities)	Are the utilities incorporated appropriate for the specific decision problem?	Yes		Yes		Yes		Yes			
Non-transparent data incorporation bias	Is the process of data incorporation transparent? Are all data and their sources described in detail?	Yes		Yes		Yes		Yes			
Limited scope bias	Have the four principles of uncertainty (methodological, structural, heterogeneity, and parameter) been considered?	Yes		Partly		Partly		Partly			
III Bias related to consistency											
Bias related to internal consistency	Has internal consistency in terms of mathematical logic been evaluated?	Unclear		Yes		Unclear		Unclear			
Type of bias	Issues addressed	Beca, 2019 [29]		Bleser, 2020 [30]		Parikh, 2020 [31]		Parmar, 2021 [32]			
		Relevant to study Yes/No/Partly/Unclear/NA	How did you deal with this bias? (description of strategy and rationale)	Relevant to study Yes/No/Partly/Unclear/NA	How did you deal with this bias? (description of strategy and rationale)	Relevant to study Yes/No/Partly/Unclear/NA	How did you deal with this bias? (description of strategy and rationale)	Relevant to study Yes/No/Partly/Unclear/NA	How did you deal with this bias? (description of strategy and rationale)		
PART A. Overall checklist for bias in economic evaluation											
Narrow perspective bias	Was a societal perspective adopted? If not, has a different perspective been justified?	Yes	Canadian public payer perspective	Yes	Health care system (NHI & patient co-payment)	Yes	US payer	Yes	Health care system		
Inefficient comparator bias	Was the best alternative chosen as a comparator? Was current practice chosen as a comparator? Have all comparators been described in sufficient detail?	Yes		Yes		Yes		Yes			
Cost measurement omission bias	Were all costs relevant to the disease and intervention identified and considered?	Yes		Yes		Yes		Yes			
Intermittent data collection bias	Was the resource use measured continuously?	Yes		Yes		Yes		Yes			
Invalid valuation bias	Is the price calculation presented in a detailed manner? Have reference prices been used?	Yes		Yes		Yes		Yes			
Ordinal ICER bias	Have cardinal scales for the outcome measure in a CEA been used?	Yes	cost/QALY; cost/LYG	Yes	cost/QALY	Yes	QALY (net monetary benefit values)	Yes	QALY, LYG		
Double-counting bias	Are variables adequately checked for double-counting?	Yes		Partly		Yes		Yes			
Inappropriate discounting bias	Have discounting rates from guidelines been applied?	Yes	1.50%	Yes	3% (cost) and 1.5% (benefits)	Yes	3%	Yes	1.50%		
Limited sensitivity analysis bias	Have the four principles of uncertainty (methodological, structural, heterogeneity, and parameter) been considered in sufficient detail?	Partly	One-way sensitivity analysis	Yes	PSA, DSA, scenarios	Yes	PSA and DSA done	Partly	Scenarios		
Sponsor bias	Have sponsorships been disclosed? Is the study protocol freely accessible?	No		Partly		Unclear		Unclear			
Reporting and dissemination bias	Has the study/trial been listed in a trial register? Have all results been reported according to the study protocol?	Unclear		Unclear		Unclear		Unclear			
PART B. Model-specific aspects of bias in economic evaluation											
I Bias related to structure											
Structural assumptions bias	Is the model structure in line with coherent theory? Do treatment pathways reflect the nature of disease?	Yes		Yes		Yes		Yes			
No treatment comparator bias	Is there an adequate comparator, i.e., care as usual?	Yes		Yes		Yes		Yes			
Wrong model bias	Is the model chosen adequate regarding the decision problem?	Yes		Yes		Yes		Yes			
Limited time horizon bias	Was a lifetime horizon chosen? Were shorter time horizons adequately justified?	Yes	15 years	Partly	5 years	Yes	10 years	Yes	Lifetime		
II Bias related to data											
Bias related to data identification	Are the methods of data identification transparent? Are all choices justified adequately? Do the input parameters come from high-quality and well-designed studies?	Yes		Partly		Yes		Yes			
Bias related to baseline data	Are probabilities, for example, based on natural history data? Is transformation of rates into transition probabilities done accurately?	Yes		Yes		Yes		Yes			
Bias related to treatment effects	Are relative treatment effects synthesized using appropriate meta-analytic techniques? Are extrapolations documented and well justified? Are alternative assumptions explored regarding extrapolation?	Yes		Partly		Yes		Yes			
Bias related to quality-of-life weights (utilities)	Are the utilities incorporated appropriate for the specific decision problem?	Yes		Yes		Yes		Yes			
Non-transparent data incorporation bias	Is the process of data incorporation transparent? Are all data and their sources described in detail?	Yes		Yes		Yes		Yes			
Limited scope bias	Have the four principles of uncertainty (methodological, structural, heterogeneity, and parameter) been considered?	Partly		Yes		Yes		Partly			
III Bias related to consistency											
Bias related to internal consistency	Has internal consistency in terms of mathematical logic been evaluated?	Unclear		Unclear		Unclear		Unclear			
